# Improvement of Gel Quality of Squid (*Dosidicus gigas*) Meat by Using Sodium Gluconate, Sodium Citrate, and Sodium Tartrate

**DOI:** 10.3390/foods11020173

**Published:** 2022-01-10

**Authors:** Yanjiao Chu, Shanggui Deng, Guancheng Lv, Mingao Li, Hongli Bao, Yuanpei Gao, Ru Jia

**Affiliations:** 1 Key Laboratory of Health Risk Factors for Seafood of Zhejiang Province, College of Food Science and Pharmacy, Zhejiang Ocean University, Zhoushan 316022, China; S19083200009@zjou.edu.cn (Y.C.); dengshanggui@zjou.edu.cn (S.D.); 18806726079@zjou.edu.cn (G.L.); Z20095135048@zjou.edu.cn (M.L.); Z20095135005@zjou.edu.cn (H.B.); 2 College of Food and Pharmaceutical Sciences, Ningbo University, Ningbo 315211, China

**Keywords:** squid surimi, myofibrillar protein, gel properties, organic salt

## Abstract

In order to improve the quality of squid surimi products, squid surimi gels were prepared using several types of organic salts under two heating conditions to study the effects of organic salts on squid gel properties. Compared with the NaCl group, organic salts reduced the solubilization capacity of myofibrillar protein, and significant (*p* < 0.05) decreases in the breaking force, breaking distance, texture, and water-holding capacity of the gel were observed in the sodium gluconate group, while significant (*p* < 0.05) increases in the breaking force, breaking distance, texture, and water-holding capacity of the gel were observed in the sodium citrate and sodium tartrate groups. Although the mixed addition of NaCl and organic salt improved surimi gel quality, the effective improvement was still lower than that of only organic salt. Rheological properties indicated that sodium citrate and sodium tartrate had high viscoelasticity. The squid surimi gel prepared by direct heating exhibited better properties than gels prepared by two-step heating. The chemical force of squid gel prepared with sodium citrate and sodium tartrate formed a stronger matrix than the gels prepared with other salts. For color, the addition of sodium citrate resulted in an undesirable color of squid surimi gels, while the addition of sodium tartrate improved the whiteness of the surimi gel. The results showed that the quality of surimi gel was dependent upon the choice of heating method and the types of salt used. Sodium citrate and sodium tartrate could significantly improve the gel properties of squid surimi. This study provides reliable guidance for improving the overall quality of squid surimi gels.

## 1. Introduction

Myofibrillar proteins (MPs) are a group of gelatinous, salt-soluble proteins accounting for 55–65% of the total meat muscle proteins [1]. High-purity MPs, also known as surimi, are usually obtained by the boning, harvesting, rinsing, and dehydration of fish and are processed into surimi-based products [2]. In common production, two types of heating methods are used to produce surimi-based products, including direct heating and two-step heating. Direct heating is performed at high temperature (90–100 °C), and two-step heating is performed at low temperature (0–40 °C) for improvement of the gelation of proteins, followed by high-temperature heating. Surimi-based products are popular because of their delicious taste, high protein, low fat, and easy storage.

According to the FAO (Food and Agriculture Organization of the United Nations), global consumption of fish grew at an average annual rate of 3.1% from 1961 to 2017, with approximately 88% (156 million tons) of total global fish production in 2018 for direct human consumption [3]. In contrast, the state of marine fisheries resources continues to deteriorate, with 34.2% of global marine fisheries being overfished in 2017; global capture fisheries production in 2018 increased by 5.4% compared to the average production of the previous three years [3]. In contrast to the decline in the population of fish, cephalopod production continues to increase worldwide due to the ability of cephalopods to rapidly adapt to changes in their surroundings and climate [4]. Doubleday et al. [4] surveyed “all major marine regions (69% northern hemisphere, 31% southern hemisphere), as well as key populations of cephalopods (52% squid, 31% octopus, 17% cuttlefish and marine phosphorus), and they found that cephalopod populations had been increased from 1953 to 2013”. Squid, the most caught cephalopods, have a high reproductive capacity and short growth cycle, which provide sufficient resources for seafood exploitation [4,5]. Squid has huge market potential in surimi production because of its high nutritional value and thick, white muscle [6].

Gel strength is an important index for evaluating the quality of surimi products. Unlike other fish, the gel made from squid is weaker due to its endogenous protease, metalloproteinases (MMPs), which are activated by Zn^2+^ to degrade heavy meromyosin (HMM) and light meromyosin (LMM) [7,8]. Therefore, activity inhibition of MMPs might provide better quality surimi-based products prepared from squid. The activity of MMPs might be inhibited by adding chelating agents such as ethylenediaminetetraacetic acid (EDTA) and 1,10-phenanthroline [8]; however, these chemical reagents are toxic and illegal for food use. Therefore, it is necessary to find food-grade additives that inhibit the hydrolysis of MPs from MMPs.

Sodium gluconate (SG), sodium citrate (SC), and sodium tartrate (ST) are strong metal chelating hydroxy carboxylic acid food additives capable of chelating most divalent and trivalent metal ions. Geng [9] reported that SC, SG, and ST inhibited the hydrolysis of squid meat protein and the degradation of MHC to varying degrees. Therefore, we speculate that the organic salts may inhibit or reduce the enzymatic activity by chelating Zn^2+^ in squid to achieve the purpose of improving the gelatinization properties of squid minced fish. Surimi products are usually supplemented with 2–3% salt to unfold protein molecules and for the dissolution of myosin. Therefore, organic salts (SG, SC, and ST) theoretically both promote the solubilization of MPs and improve the properties of squid gels by inhibiting the activity of MMPs.

In this study, organic salts (SG, SC, and ST) were selected to prepare squid surimi gels in an effort to completely or partially replace NaCl. By considering the solubility of salt to MPs and the suitability of salt flavor, 2.5% of salt was used to prepare samples, and direct heating and two-step heating were used to prepare surimi gel. Finally, the effects of salt substitution and heating conditions on the gel properties of squid surimi were studied.

## 2. Materials and Methods

### 2.1. Materials and Reagents

Peruvian squid (*Dosidicus gigas*) (average length: 61.04 ± 7.21 cm; average weight: 383.09 ± 131.08 g) were purchased from Shenjiamen Fishing Market at Zhoushan City in Zhejiang Province, China. Samples were transported to the laboratory on ice. The head, tail, and skin were removed, and mantles were collected and stored in a refrigerator (MDF–U53V, Sanyo Corporation, Osaka, Japan) at −80 °C for later use. Proximate compositions were determined according to the Chinese national standards for food safety, the content moisture [10], crude lipid [11], and ash [12] were 77.14 ± 0.46%, 1.08 ± 0.03%, and 1.52 ± 0.05%, respectively. The crude protein was determined by the biuret method [13], and the content was 15.85 ± 0.07%. Sodium chloride, sodium citrate, copper (II) sulfate pentahydrate, potassium sodium tartrate tetrahydrate, potassium Iodide, sodium hydroxide, urea, trichloroacetic acid (TCA), and β-mercaptoethanol were purchased from China Shanghai Sinopharm Chemical Reagent Co., Ltd.(Sinopharm Chemical, Shanghai, China); SG and ST were purchased from Shanghai Aladdin Biochemical Technology Co., Ltd. (Aladdin Biochemical Technology, Shanghai, China).

### 2.2. Solubility of Squid Muscle Protein

The solubility of squid protein was determined according to the method described by Gao [14], with slight modifications. Approximately 1 g of squid muscle was homogenized with 20 mL of 0.6 M salt solution by a homogenizer (T18 digital, IKA Group, Staufen, Germany) at 10,000 rpm for 30 s. The mixture was then centrifuged at 13,000× *g* (Multifuge X1R, Thermo Fisher Scientific Co., Ltd., Shanghai, China) at 4 °C for 20 min. The protein concentration of the supernatant was determined by the biuret method. Solubility was expressed as the ratio of the protein content dissolved in organic salt to the protein content dissolved in NaCl.

### 2.3. Preparation of Squid Surimi Paste and Gel

Squid mantles were cut into small pieces and chopped in a food processor (FP 3010, Braun (Shanghai) Co., Ltd., Shanghai, China) for 1 min. Then, 2.5% salt (NaCl, SG, SC, ST, NG, NC, and NT; where NG, NC, and NT were the mixtures of NaCl with SG, SC, and ST, respectively; NaCl and organic salt were in concentrations of 1.25% each) was added, and the mantle pieces were further chopped for 3 min. Finally, ice water was added to adjust the moisture content to 78%. During this period, the temperature of the surimi ointment was kept below 10 °C. The surimi ointment was packed in a plastic shell (2.5 cm in diameter) and heated by either direct heating (90 °C, 30 min) or two-step heating (40 °C for 30 min, followed by 90 °C for 30 min). After heating, the gel samples were stored at 4 °C.

### 2.4. Determination of Rheological Properties

The rheological properties were determined according to the method described by Xue [15], with slight modifications. The rheological properties of surimi ointment prepared with different salts were measured using a rheometer (HR-20, Waters Technology (Shanghai) Co., Ltd., Shanghai, China). The test parameters were as follows: the temperature scanning mode was adopted, the oscillation frequency was 1 Hz, the strain was 2%, the distance between parallel plates was 1 mm, the heating scanning range was 20–90 °C, the heating rate was 4 °C/min, and the changes in storage modulus (*G′*) and loss modulus (*G″*) during heating were measured.

### 2.5. Texture Analysis

The texture of the gel was determined by a texture analyzer (TMS-PRO, FTC USA, Inc., Sterling, VA, USA). In this study, tests included gel strength and textural profile. The measurements were performed on a cylindrical piece of gel with 2.5 cm of height and 2.5 cm of diameter.

Breaking strength was determined using a 5-mm spherical metal probe. The results were recorded as breaking force (N) and breaking distance (mm). The specific parameters were as follows: 5 mm diameter of the spherical plunger, 20 N of the load cell, and 1 mm/s of depression speed.

The texture profile analysis was measured with a cylindrical probe P/250 upon two-cycle compression tests. The initial force of the physical property tester was 0.6 N. The detection speed was 1 mm/s. The deformation was 30% of sample height. Texture parameters, such as hardness, adhesiveness, cohesiveness, springiness, and chewiness, of the sample, were obtained.

### 2.6. Water-Holding Capacity (WHC)

The measurement of water-holding capacity (WHC) was carried out according to the method described by Chaijan [16], with a slight modification. Approximately 1 g of surimi gel was cut into 5-mm-thick slices and marked as *M*_1_. The sample was wrapped in three layers of filter paper and placed in a 50-mL centrifuge tube. Then, the sample was centrifuged at a speed of 8000× *g* (Multifuge X1R, Thermo Fisher Scientific (China) Co., Ltd., Shanghai, China) at 4 °C for 15 min. The mass of the sample after centrifugation was recorded as *M*_2_. The WHC was calculated by using the following equation:

(1) $$\mathrm{WHC}=\frac{M_{2}}{M_{1}}\times 100\%$$

### 2.7. Determination of Whiteness

The luminance (L*), redness (a*), and yellowness value (b*) of the cross-sections were detected by using a Colorimeter (CS-210, Hangzhou Chnspec Technology Co., Ltd., Hangzhou, China). The whiteness was calculated using the following formula.

(2) $$\mathrm{Whiteness}=100-[{\left(100-L^{*}\right)}^{2}+a^{*2}+b^{*2}]$$

### 2.8. Determination of Chemical Forces

The method proposed by Gómez-Guillén and Tan [17,18] was referred to for determining chemical forces, with some modifications. The surimi dissolving liquids were S_1_ (0.05 M NaCl), S_2_ (0.6 M NaCl), S_3_ (0.6 M NaCl + 1.5 M urea), S_4_ (0.6 M NaCl + 8 M urea), and S_5_ (0.6 M NaCl + 8 M urea + 0.5 M β-mercaptoethanol). About 2 g of gel sample and 10 mL of S_1_–S_4_ solution were homogenized at 8000 rpm for 30 s with a homogenizer (T18 digital, IKA Group, Staufen, Germany) and placed at 4 °C for 1 h. The mixture was centrifuged at 8000× *g* for 10 min (4 °C). The supernatant was collected and stored at 4 °C for determination of protein content. In addition, S_5_ was added to the precipitate after S_4_ treatment, and the above operation was repeated, and the supernatant was collected. The protein in the collected supernatant was rinsed with an equal volume of 20% TCA and dissolved with 1 mL of NaOH (1 M). The protein concentration was determined by the biuret method. The results were expressed in terms of the amount of protein dissolved per liter of solution (g/L). The concentrations (C) of ionic bonds, hydrogen bonds, hydrophobic interactions, and disulfide bonds were calculated as follows:

Ionic bond = C_S2_ − C_S1_

Hydrogen bond = C_S3_ − C_S2_

Hydrophobic interaction = C_S4_ − C_S3_

Disulfide bond = C_S5_ (3)

### 2.9. Data Analysis

Three independent replicate tests were conducted for each group of samples. The results were expressed as mean ± standard deviation of three replicates. The protein solubility of squid muscle was analyzed by one-way ANOVA and Duncan method, and other test data were analyzed using two-way ANOVA and LSD method in SPSS 26.0 (SPSS Inc., Chicago, IL, USA). A value of *p* < 0.05 was considered to be statistically significant.

## 3. Results and Discussion

### 3.1. Solubility of Squid Protein

**Figure 1:**
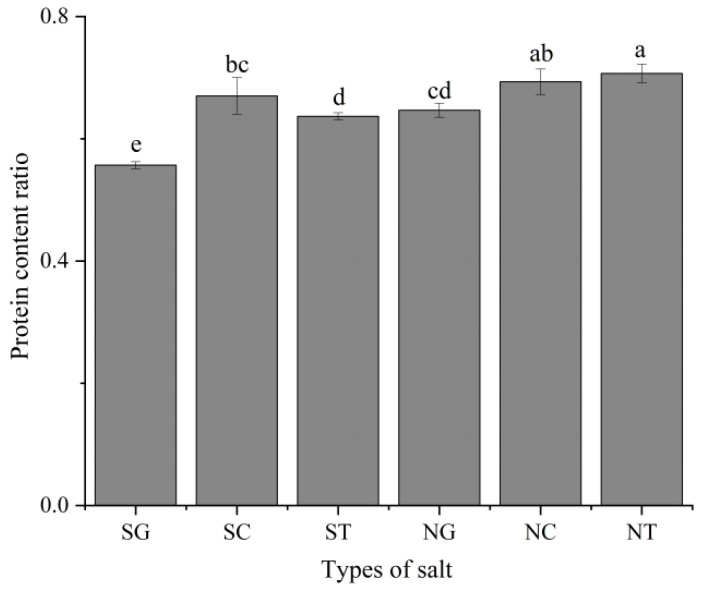
Solubility of different types of salt for protein. Values are mean ± standard deviation (*n* = 3). Different letters represent significant differences (*p* < 0.05). SG: Sodium gluconate; SC: sodium citrate; ST: sodium tartrate; NG: NaCl + Sodium gluconate; NC: NaCl + sodium citrate; NT: NaCl + sodium tartrate.

The solubility of squid muscle proteins was the ratio of the protein content dissolved in the tested organic salts to the protein content dissolved in NaCl (Figure 1). Protein solubility was significantly different for different types of organic salts (*p* < 0.05). The protein solubility of samples treated with organic salts was lower than that treated with NaCl (NaCl is used as 1). Among them, the protein solubility of the SG group was the lowest, while the protein solubility of the SC group was the highest. The protein solubility of NG and NT was significantly higher than that of SG and ST (*p* < 0.05). Tahergorabi [19] reported that the ability of Cl^−^ to dissolve MPs was greater than that of Na^+^. Therefore, the increase of solubility may be due to the presence of NaCl, which increases the dissolution of MP. Salt-soluble (>0.3 M) proteins are mainly MPs, myosin, and actin, all of which promote gel-forming [20]. Therefore, these results were used as an important basis for determining the ability of different organic salts to form gels on surimi.

### 3.2. Rheological Properties of Surimi Ointment

**Figure 2:**
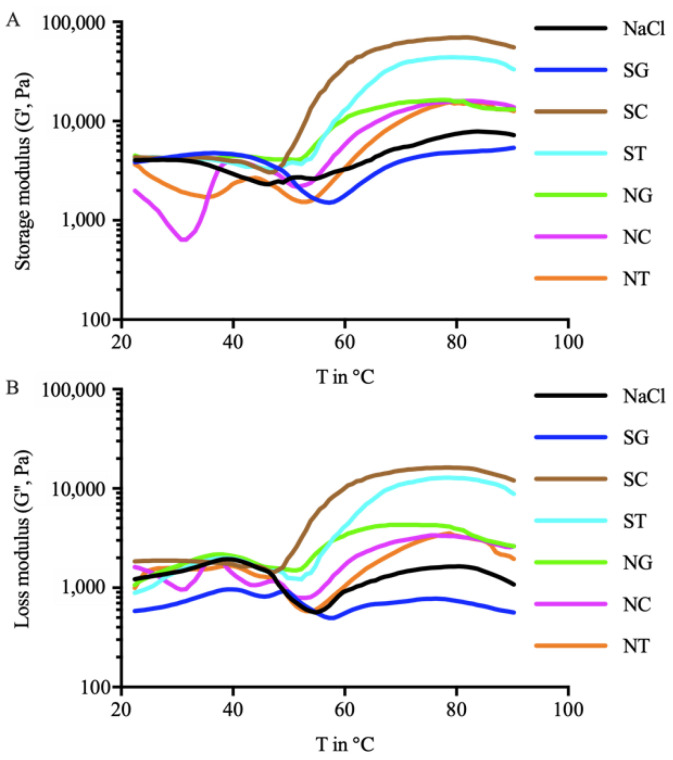
Rheological properties of surimi paste treated with different organic salts, where (**A**) represents the storage modulus (*G′*) and (**B**) represents the loss modulus (*G″*) (n = 3). SG: Sodium gluconate; SC: sodium citrate; ST: sodium tartrate; NG: NaCl + Sodium gluconate; NC: NaCl + sodium citrate; NT: NaCl + sodium tartrate.

Dynamic rheological properties were simulated for the gelling process of surimi ointment at 20–90 °C for different treatment groups (Figure 2). The changes in *G′* and *G″* vary across different groups. First, the *G′* of NaCl (29–46 °C), SC (20–26 °C), NC (20–33 °C), and NT (22–36 °C) decreased in varying degrees from 20 °C to 46 °C (Figure 2A). Liu [21] hypothesized that an increase in temperature might increase the fluidity of myosin and affect the aggregation between MPs, resulting in a slight decrease in *G′*. The significant decrease of *G′* and *G″* in the NC group from 22 °C to 31 °C might be due to the excessive oxidation of MPs caused by the addition of SC and NaCl [1,22]. The *G′* of NaCl (46–48 °C), SG (20–36 °C), SC (26–32 °C), ST (43–50 °C), NG (35–41 °C), NC (33–39 °C), and NT (36–43 °C) increased slightly in the temperature range of 20 °C–50 °C (Figure 2A), which was the result of the denaturation and aggregation of the myosin head and cross-linking components, and indicated the initial formation of a gel network [23]. Then NaCl (48–54 °C), SG (36–57 °C), SC (32–47 °C), ST (50–53 °C), NG (41–51 °C), NC (39–51 °C), and NT (43–52 °C) groups deteriorated at 32–57 °C. Qin [24] reported that the rate of bond breakage caused by protein denaturation exceeded the rate of bond formation, which indicated the increase of thermal denaturation and the rearrangement of protein molecules. Liu [25] confirmed that the decrease of *G′* might be due to the high activity of endogenous proteolytic enzymes in muscles in this temperature range, which resulted in the degradation of MPs. Afterward, *G′* continued to increase and stabilize. Compared with the NaCl group, organic salt groups completed the initial formation and deterioration of the surimi gel at a lower temperature, indicating that the organic salts were easier to promote the gelation of the squid surimi. Throughout the heating process, *G′* was always larger than *G″*, indicating that *G′* was dominant in the gelation process. The high *G′* of SC and ST indicated that organic salts SC and ST had a positive effect on the formation of surimi gels.

### 3.3. Texture Analysis

#### 3.3.1. Breaking Strength of Gels

**Figure 3:**
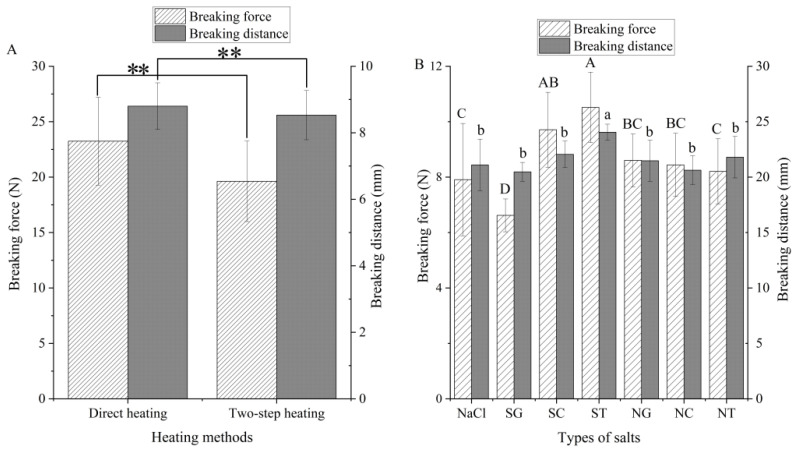
Breaking force and breaking distance of gels prepared by different heating method (**A**) and salt (**B**)**.** Values are mean ± standard deviation (heating methods: *n* = 21; types of salts: *n* = 6). ** represents the significant difference (*p* < 0.01). Different letters indicate significant differences (*p* < 0.05). SG: Sodium gluconate; SC: sodium citrate; ST: sodium tartrate; NG: NaCl + Sodium gluconate; NC: NaCl + sodium citrate; NT: NaCl + sodium tartrate.

Generally, the breaking distance reflected the degree of deformation of the gel and had a positive correlation with gel elasticity [26]. In this study, both heating method and salt type independently and significantly (*p* < 0.05) affected the breaking force of gel, while the effect of the interaction of the two factors (type of salt and heating method) was not significant (*p* > 0.05) (data are shown in the Tables S1 and S2). Moreover, the breaking distance was only significantly (*p* < 0.05) affected by the salt type. The breaking force and breaking distance of the gels treated with direct heating were higher than gels treated with two-step heating (Figure 3A). This result was different from the general cognitive, which claimed that surimi gels heated via the two-step method had breaking strength [27]. Okamoto [8] found that the optimum temperature of MMPs (myosin I and myosin II) that hydrolyzed the MHC in squid was 40 °C. In this study, the temperature of 40 °C was used for two-step heating; therefore, MMPs hydrolyzed MPs resulted in the decline of the quality of surimi products prepared by two-step heating.

The addition of SC and ST improved the breaking force and breaking distance of gel compared to the addition of NaCl. Although the addition of NaCl increased the dissolution of MPs, NaCl might reduce the inhibition of organic salt on the activity of MMPs, which partially hydrolyzed the MPs and resulted in decreased gel quality. The breaking force of the SG group was lower than that of the NaCl group (*p* < 0.05). This result indicated that the low solubility of MPs in squid flesh led to a reduction in the cross-linking of the three-dimensional gel network dependent on the heating of MPs, consequently, decreased the gel quality in the SG group. The breaking distance and breaking force of gels treated with mixed salts were similar to those treated with only NaCl, and the phenomenon might be due to the low content of organic salts decreased inhibition of the activity of MMPs. The results indicated that the heating method and the type of organic salt affected the gel properties of squid surimi gels.

#### 3.3.2. Texture Profile Analysis

Both the heating method and the type of salt had significant effects on the texture of the surimi gel (Table 1). Texture parameters, except hardness and chewiness, were not affected by the interaction of two factors (data are shown in the Tables S3–S7). Compared to the two-step heating, the direct heating samples had higher hardness, cohesiveness, chewiness, and springiness. The higher cohesiveness of the samples with direct heating indicated that the sample gel system was better than that with the two-step heating group. The addition of different kinds of salts also affected the texture of the surimi gel. Hardness adhesiveness and chewiness of the SG group were significantly lower than those of the NaCl group (*p* < 0.05) due to SG’s poor ability to dissolve MPs, which led to a weaker surimi gel-forming ability and corresponded lower breaking force. However, the hardness and chewiness of the SC group were significantly higher than those of the NaCl group. This was consistent with the conclusion that SC increased the hardness and chewiness of surimi, previously reported by Filomena-Ambrosio [28]. Ruusunen [29] reported that SC improved the texture of meat by increasing the value of pH. Therefore, there are two possible reasons for SC to improve the quality of surimi gels: shifting of the pH and inhibition of MMPs activity. The mixed salt group showed reduced adhesiveness compared to samples with single salt addition. The springiness of surimi gels was not significantly affected by salt types and heating methods (*p* > 0.05).

In the case of interaction of two factors, the hardness and chewiness of the surimi gel under direct heating were significantly higher (*p* < 0.05) compared to those of surimi gels subjected to two-step heating (Table 2). Compared to the NaCl group, the SC group had higher hardness and chewiness under both heating methods. Under direct heating, mixed salt could not improve the hardness and chewiness of gel compared to the NaCl group, while the NC group significantly improved the hardness and chewiness of gel under two-step heating.

### 3.4. Water-Holding Capacity (WHC)

**Figure 4:**
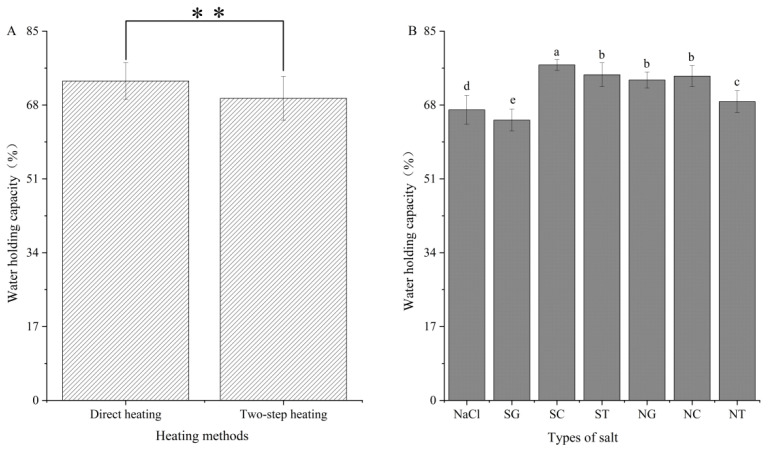
Water holding capacity of gels prepared by different heating method (**A**) and salt (**B**). Values are mean ± standard deviation (heating methods: *n* = 21; types of salts: *n* = 6). ** represents the significant difference (*p* < 0.01); different letters indicate significant differences (*p* < 0.05) in salt types. SG: Sodium gluconate; SC: sodium citrate; ST: sodium tartrate; NG: NaCl + Sodium gluconate; NC: NaCl + sodium citrate; NT: NaCl + sodium tartrate.

WHC of gels was affected by only heating method and salt type. (*p* < 0.05) (data are shown in the Table S8). The WHC of surimi gel under direct heating was significantly higher than that with two-step heating (*p* < 0.05) (Figure 4A). Generally, the result of WHC was similar to the breaking force. In addition, the WHCs of gels prepared with organic salts were higher than those of gels prepared with NaCl, except SG. It was reported that SG, SC, and ST increased the negative charge of MPs and promoted electrostatic repulsion between MPs, resulting in improved WHC for surimi gels [30]. Kuwahara [31] reported the ability of SC to retain water and prevent actomyosin degeneration. SG, SC, and ST might also enhance gel quality by inhibiting actomyosin denaturation, thereby improving WHC. However, in the present study, the WHC of the SG group was lower than that of the other groups, possibly due to its lower MPs dissolution ability. Moreover, due to the addition of NaCl, the solubility of the mixed salt NG was significantly improved, which improved the WHC of the NG surimi gel group. This further demonstrated the importance of MPs concentration for gel formation. Although the MPs solubility of the NC and NT groups was improved to a certain extent due to the addition of NaCl (Figure 1), the WHC of the two groups of surimi gels was reduced compared to their single organic salt components. One possible explanation was that the addition of NaCl led to a reduction in organic salts, which weakened the inhibition of organic salts on the activity of MMPs and led to the degradation of MPs and reduced WHC. Holmer [32] used SC instead of NaCl in saline solution in the study on the palatability of cow muscles and found that the WHC of the muscles treated with the SC solution was not changed, and the pH value of the muscles treated with the SC solution was significantly higher than that of cow muscles treated with NaCl. Ruusunen [29] reported that SC improved the texture by increasing the pH value, thereby increasing the WHC of the sausages in their research. Although the effect of ST on surimi gel had rarely been reported, its metal chelating ability was similar to that of SC, and its inhibitory effect on the degradation of MHC provided the possibility to improve the WHC of squid surimi.

### 3.5. Whiteness Analysis

**Figure 5:**
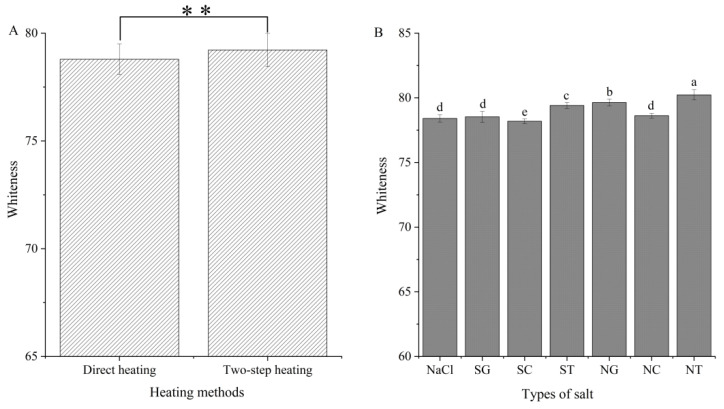
Whiteness of gels prepared by different heating method (**A**) and salt (**B**). Values are mean ± standard deviation (heating methods: *n* = 21; types of salts: *n* = 6). ** represents the significant difference (*p* < 0.01); different letters indicate significant differences (*p* < 0.05) in salt types. SG: Sodium gluconate; SC: sodium citrate; ST: sodium tartrate; NG: NaCl + Sodium gluconate; NC: NaCl + sodium citrate; NT: NaCl + sodium tartrate.

Whiteness is an important sensory index to measure the quality of surimi gel. It was found that the heating method and the type of salt had a significant effect on the whiteness of the gel (*p* < 0.05). However, the interaction between the heating method and salt type was not significant (*p* > 0.05) (data are shown in the Table S9). Except for SC (78.19 ± 0.19), the whiteness of gel with the addition of organic salts was higher than that of the NaCl (78.41 ± 0.28) group. SG, SC, and ST inhibited the activity of MMPs and slowed down the autolysis of squid, thus reducing the production of peptides and free amino acids that caused the Maillard reaction [9]. Therefore, the increase in gel whiteness of surimi prepared using SG, ST, NG, and NT might be due to the inhibition of the Maillard reaction during heating [33]. Holmer [32] found that the color of steaks treated with SC solution was darker than the color of steaks treated with NaCl solution because SC had a higher pH than NaCl, which resulted in less light reflection and darker color. The addition of SC did not significantly affect the whiteness of the surimi gel due to changes in pH. The whiteness of the mixed salts NG, SC, and NT group was also improved compared with either single organic salt SG, SC, or ST. This was because the addition of NaCl promoted the dissolution of MPs and formed more network cross-linking between MPs, which improved light transmittance. In addition, the whiteness of surimi gels containing ST was significantly higher than that of the gels containing the other two salts (*p* < 0.05). Endo [34] reported that ST was particularly effective in slowing down the thermal degradation of vegetable oils, reducing the hydrolysis, thermal oxidation, polymerization, and coloration of vegetable oils when heated at high temperatures. Therefore, the inhibition of the coloration produced by the heating process of surimi ointment by the ST on the Maillard reaction and thermal degradation of oil might be the reason for the higher whiteness of gels. In addition, two-step heating had a higher gel whiteness than direct heating (*p* < 0.05) (Figure 5A). The two-step heating exposed more chemical groups to participate in the formation of the gel network than the direct heating, which increased light transmittance.

### 3.6. Analysis of Chemical Forces

**Figure 6:**
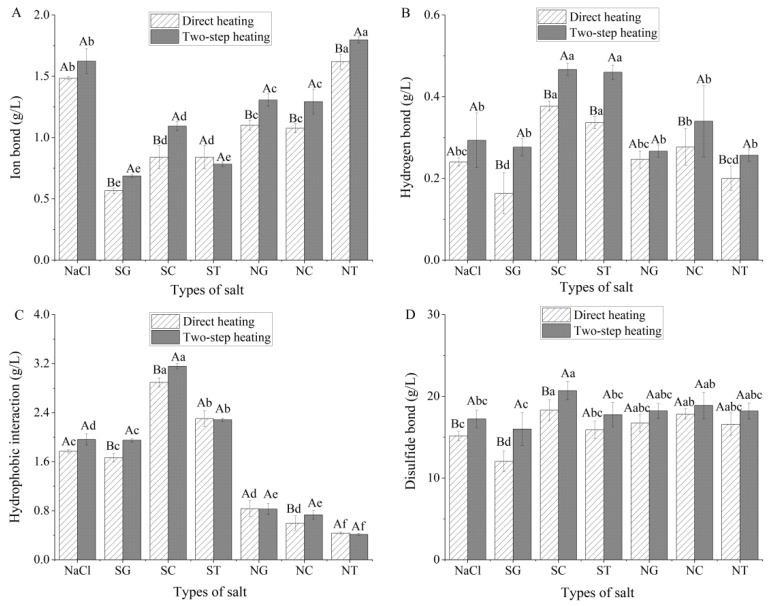
Chemical analysis of squid surimi gel prepared using different organic salts and heating methods (*n* = 3). Content of ion bond, hydrogen bond, hydrophobic interaction, and disulfide bond were showed in the (**A**–**D**), respectively. Values are mean ± standard deviation. Different capital letters indicate significant differences (*p* < 0.05) between heating methods; lower case letters indicate significant differences (*p* < 0.05) in salt types. SG: Sodium gluconate; SC: sodium citrate; ST: sodium tartrate; NG: NaCl + Sodium gluconate; NC: NaCl + sodium citrate; NT: NaCl + sodium tartrate.

The chemical forces in surimi gels mainly include ionic bonds, hydrogen bonds, hydrophobic interactions, and disulfide bonds. From the results, ionic bond and hydrophobic interaction were significantly (*p* < 0.05) affected by not only heating method and salt type but also interaction of two factors (data are shown in the Tables S10–S13). Compared with direct heating, two-step heating surimi formed more chemical forces (Figure 6) because the 40 °C heating portion of the two-step method allowed the protein to expand slowly and expose more groups in MPs. Compared with ionic bonds and hydrogen bonds, surimi gels processed via the two-step method contained a higher hydrophobic interaction force (Figure 6A–C). Wang [35] believed that the hydrophobic interaction was higher than other molecular forces because heating opened the protein structure, exposed hydrophobic amino acids and sulfhydryl groups to a polar environment, and induced the binding of MPs molecules through hydrophobic forces and disulfide bonds. Zhang [36] reported that hydroxyl groups competed for the hydrophilic group of gel proteins and promoted the hydrophobic interaction of intermolecular and intramolecular surimi gels. Although SG contained more hydroxyl groups than SC and ST, the chemical groups between MPs were not fully exposed during heating due to the lower solubility of MPs, which affected the formation of chemical forces. In addition, the hydrophobic interaction contents of the NC and NT groups were lower than those of the NaCl control group and similar single organic salts (*p* < 0.05), possibly due to the reduction in hydroxyl groups, and consequently, the competition of hydroxyl groups with hydrophilic groups in gel proteins was reduced. Liu [37] reported that the aggregation of hydrophobic groups significantly increased the compactness of the surimi gel network structure, which in turn affected the characteristics of the surimi gel. The lower hydrophobic force of the SG group and the higher hydrophobic force of the SC and ST groups were also consistent with the results of the breaking force, texture characteristics, and WHC of SG, SC, and ST. Under direct heating, the disulfide bonds of the SG, SC, and NC groups differed significantly from those of the NaCl group (*p* < 0.05) (Figure 6D). The changes in disulfide and hydrogen bonds were similar to the changes of gel breaking force (Figure 3A), and it was speculated that the formation of surimi gel depended on hydrogen and disulfide bonds. However, the breaking distance of the gels did not exactly coincide with the changes in disulfide and hydrogen bonds, indicating that hydrogen and disulfide bonds were not the main chemical forces maintaining the stable conformation of surimi gels. This was in agreement with the conclusion of Gómez-Guillén [17], who reported that hydrogen bonding did not maintain the stability of the conformation of surimi gels.

## 4. Conclusions

In order to alleviate the shortcomings of the insufficient gelling capacity of squid surimi available in the market, this study focused on utilizing organic salts to improve the quality of squid surimi products. Two different heating methods were used to reveal the effects of organic salts on gel formation ability and the quality of surimi gel. The results showed that compared to NaCl, organic salts reduce the solubilization capacity of myofibrillar protein; however, compared to gels treated with NaCl, the addition of SC and ST increased the breaking force, breaking distance, texture characteristics, and WHC of surimi gel. The results of the chemical forces further explained the effect of different organic salts on the gel properties of surimi gel. On the other hand, the addition of organic salts darkened the color of the gel and weakened the solubility of MPs. Generally, organic salts SC and ST improved the gel properties of squid by inhibiting the degradation of MPs from improving the product quality. In addition, the low-temperature heating temperature of 40 °C used for the two-step heating in this study resulted in a higher metalloprotease enzyme activity in the squid, making the gelation properties of the two-step heated surimi lower than those of direct heating.

## Figures and Tables

**Table 1:** Analysis of main effects of gel texture of minced squid.

	Treatment	Hardness (N)	Adhesiveness (mJ)	Cohesiveness (Ratio)	Springiness (mm)	Chewiness (mJ)
Heating methods	Direct heating	9.99 ± 1.41 A	0.19 ± 0.09 A	0.64 ± 0.02 A	5.57 ± 0.31 A	35.20 ± 5.9 A
	Two-step heating	8.76 ± 1.25 B	0.21 ± 0.09 A	0.61 ± 0.03 B	5.40 ± 0.26 A	28.86 ± 5.48 B
Types of salt	NaCl	9.84 ± 1.52 b	0.26 ± 0.10 ab	0.62 ± 0.03 b	5.61 ± 0.38 ab	34.25 ± 7.33 a
	SG	7.74 ± 0.89 c	0.17 ± 0.06 c	0.61 ± 0.05 b	5.26 ± 0.24 b	25.11 ± 5.00 c
	SC	11.24 ± 0.78 a	0.32 ± 0.06 a	0.63 ± 0.03 ab	5.64 ± 0.21 a	39.72 ± 3.79 b
	ST	9.46 ± 0.71 b	0.21 ± 0.05 bc	0.61 ± 0.01 b	5.56 ± 0.17 ab	32.27 ± 3.80 b
	NG	9.31 ± 1.36 b	0.14 ± 0.08 c	0.63 ± 0.02 ab	5.48 ± 0.21 ab	31.97 ± 6.46 b
	NC	9.86 ± 1.07 b	0.16 ± 0.03 c	0.65 ± 0.02 a	5.57 ± 0.43 ab	34.10 ± 3.08 b
	NT	8.19 ± 0.77 c	0.14 ± 0.05 c	0.62 ± 0.01 b	5.32 ± 0.23 ab	26.80 ± 3.53 c

Values are mean ± standard deviation (heating methods: *n* = 21; types of salts: *n* = 6). Different capital letters indicate significant differences (*p* < 0.05) between heating methods; lower case letters indicate significant differences (*p* < 0.05) in salt types. SG: Sodium gluconate; SC: sodium citrate; ST: sodium tartrate; NG: NaCl + Sodium gluconate; NC: NaCl + sodium citrate; NT: NaCl + sodium tartrate.

**Table 2:** The hardness and chewiness of surimi gel under the influence of different heating methods and organic salts.

Treatment	Hardness (N)		Chewiness (mJ)	
	Direct Heating	Two-Step Heating	Direct Heating	Two-Step Heating
NaCl	11.20 ± 0.43 Aab	8.48 ± 0.31 Bc	40.25 ± 3.56 Aab	28.25 ± 3.71 Bc
SG	8.52 ± 0.14 Acd	6.96 ± 0.36 Bd	29.31 ± 1.45 Ade	20.90 ± 2.71 Bd
SC	11.89 ± 0.38 Aa	10.59 ± 0.32 Ba	42.83 ± 2.00 Aa	36.62 ± 1.75 Ba
ST	9.96 ± 0.38 Abc	8.97 ± 0.62 Abc	35.24 ± 1.78 Abc	29.30 ± 2.54 Bbc
NG	10.37 ± 0.41 Ab	8.26 ± 1.04 Bc	37.44 ± 2.54 Abc	26.48 ± 2.79 Bc
NC	9.77 ± 1.69 Abc	9.94 ± 0.11 Aab	34.39 ± 3.88 Acd	33.81 ± 2.90 Aab
NT	8.26 ± 0.96 Ad	8.12 ± 0.75 Ac	26.96 ± 3.98 Ae	26.66 ± 3.91 Ac

Values are mean ± standard deviation (n = 3). Different capital letters indicate significant differences (*p* < 0.05) between heating methods (same type of salt); lower case letters indicate significant differences (*p* < 0.05) in salt types (same heating method). SG: Sodium gluconate; SC: sodium citrate; ST: sodium tartrate; NG: NaCl + Sodium gluconate; NC: NaCl + sodium citrate; NT: NaCl + sodium tartrate.

## Data Availability

The data that support the findings of this study are available from the corresponding author upon reasonable request.

## Supplementary Materials

The following supporting information can be downloaded at: https://www.mdpi.com/article/10.3390/foods11020173/s1. Table S1: Tests of Between-Subjects Effects, Dependent Variable: Breaking force; Table S2. Tests of Between-Subjects Effects, Dependent Variable: Breaking distance; Table S3. Tests of Between-Subjects Effects, Dependent Variable: Hardness; Table S4. Tests of Between-Subjects Effects, Dependent Variable: Adhesiveness; Table S5. Tests of Between-Subjects Effects, Dependent Variable: Cohesiveness; Table S6. Tests of Between-Subjects Effects, Dependent Variable: Springiness; Table S7. Tests of Between-Subjects Effects, Dependent Variable: Chewiness; Table S8. Tests of Between-Subjects Effects, Dependent Variable: Water-holding capacity; Table S9. Tests of Between-Subjects Effects, Dependent Variable: Whiteness; Table S10. Tests of Between-Subjects Effects, Dependent Variable: Ionic bonds; Table S11. Tests of Between-Subjects Effects, Dependent Variable: Hydrogen bonds; Table S12. Tests of Between-Subjects Effects, Dependent Variable: Hydrophobic interactions; Table S13. Tests of Between-Subjects Effects, Dependent Variable: Disulfide bonds.
