# Supplementary material for: Improvement of Gel Quality of Squid (Dosidicus gigas) Meat by Using Sodium Gluconate, Sodium Citrate, and Sodium Tartrate
Source: Foods. 2022 Jan 10;11(2):173. doi: 10.3390/foods11020173 (PMC8775022; doi:10.3390/foods11020173)
Supplement: Supplementary file 1 [file foods-11-00173-s001.zip › foods-1514262-supplementary.pdf]

Table S1. Tests of Between-Subjects Effects

Dependent Variable: Breaking force

Source	Type III Sum of Squares	df	Mean Square	F	Sig
Corrected Model	530.958[a]	13	40.843	6.215	.000
Intercept	19285.196	1	19285.196	2934.657	.000
Heating method	137.757	1	137.757	20.963	.000
salt	355.440	6	59.240	9.015	.000
Heating method * salt	37.762	6	6.294	.958	.471
Error	184.003	28	6.572		
Total	20000.157	42			
Corrected Total	714.961	41			

a. R Squared = .743（Adjusted R Squared = .623）

Table S2. Tests of Between-Subjects Effects

Dependent Variable: Breaking distance

Source	Type III Sum of Squares	df	Mean Square	F	Sig
Corrected Model	11.935[a]	13	.918	2.634	.015
Intercept	3151.201	1	3151.201	9040.639	.000
Heating method	.752	1	.752	2.157	.153
salt	8.308	6	1.385	3.973	.005
Heating method * salt	2.875	6	.479	1.375	.259
Error	9.760	28	.349		
Total	3172.896	42			
Corrected Total	21.695	41			

a. R Squared = .550（Adjusted R Squared = .341）

Table S3. Tests of Between-Subjects Effects

Dependent Variable: Hardness

Source	Type III Sum of Squares	df	Mean Square	F	Sig
Corrected Model	73.444[a]	13	5.650	11.636	.000
Intercept	3692.906	1	3692.906	7606.025	.000
Heating method	15.959	1	15.959	32.870	.000

salt	48.017	6	8.003	16.483	.000
Heating method * salt	9.468	6	1.578	3.250	.015
Error	13.595	28	.486		
Total	3779.945	42			
Corrected Total	87.039	41			

a. R Squared = .844（Adjusted R Squared = .771）

Table S4. Tests of Between-Subjects Effects

Dependent Variable: Adhesiveness

Source	Type III Sum of Squares	df	Mean Square	F	Sig
Corrected Model	.194[a]	13	.015	3.496	.003
Intercept	1.640	1	1.640	383.794	.000
Heating method	.003	1	.003	.690	.413
salt	.173	6	.029	6.743	.000
Heating method * salt	.018	6	.003	.716	.640
Error	.120	28	.004		
Total	1.954	42			
Corrected Total	.314	41			

a. R Squared = .619（Adjusted R Squared = .442）

Table S5. Tests of Between-Subjects Effects

Dependent Variable: Cohesiveness

Source	Type III Sum of Squares	df	Mean Square	F	Sig
Corrected Model	.022[a]	13	.002	4.217	.001
Intercept	16.319	1	16.319	41288.699	.000
Heating method	.010	1	.010	26.241	.000
salt	.008	6	.001	3.177	.017
Heating method * salt	.004	6	.001	1.586	.188
Error	.011	28	.000		
Total	16.352	42			
Corrected Total	.033	41			

a. R Squared = .662（Adjusted R Squared = .505）

Table S6. Tests of Between-Subjects Effects

Dependent Variable: Springiness

Source	Type III Sum of Squares	df	Mean Square	F	Sig
Corrected Model	1.312[a]	13	.101	1.259	.293
Intercept	1265.665	1	1265.665	15793.545	.000
Heating method	.305	1	.305	3.808	.061
salt	.782	6	.130	1.627	.177
Heating method * salt	.225	6	.037	.467	.827
Error	2.244	28	.080		
Total	1269.220	42			
Corrected Total	3.556	41			

a. R Squared = .369（Adjusted R Squared = .076）

Table S7. Tests of Between-Subjects Effects

Dependent Variable: Chewiness

Source	Type III Sum of Squares	df	Mean Square	F	Sig
Corrected Model	1475.825[a]	13	113.525	13.094	.000
Intercept	43091.240	1	43091.240	4970.332	.000
Heating method	421.673	1	421.673	48.638	.000
salt	862.544	6	143.757	16.582	.000
Heating method * salt	191.608	6	31.935	3.683	.008
Error	242.751	28	8.670		
Total	44809.817	42			
Corrected Total	1718.577	41			

a. R Squared = .859（Adjusted R Squared = .793）

Table S8. Tests of Between-Subjects Effects

Dependent Variable: Water-holding capacity

Source	Type III Sum of Squares	df	Mean Square	F	Sig
Corrected Model	1003.808[a]	13	77.216	72.642	.000
Intercept	214919.039	1	214919.039	202189.297	.000
Heating method	167.560	1	167.560	157.636	.000

salt	822.293	6	137.049	128.931	.000
Heating method * salt	13.955	6	2.326	2.188	.074
Error	29.763	28	1.063		
Total	215952.609	42			
Corrected Total	1033.571	41			

a. R Squared = .971（Adjusted R Squared = .958）

Table S9. Tests of Between-Subjects Effects

Dependent Variable: Whiteness

Source	Type III Sum of Squares	df	Mean Square	F	Sig
Corrected Model	23.030[a]	13	1.772	58.090	.000
Intercept	262147.898	1	262147.898	8596247.844	.000
Heating method	1.976	1	1.976	64.808	.000
salt	20.892	6	3.482	114.179	.000
Heating method * salt	.161	6	.027	.882	.521
Error	.854	28	.030		
Total	262171.781	42			
Corrected Total	23.883	41			

a. R Squared = .964（Adjusted R Squared = .948）

Table S10. Tests of Between-Subjects Effects

Dependent Variable: Ionic bonds

Source	Type III Sum of Squares	df	Mean Square	F	Sig
Corrected Model	5.736[a]	13	.441	122.000	.000
Intercept	55.614	1	55.614	15377.149	.000
Heating method	.239	1	.239	66.155	.000
salt	5.403	6	.900	248.969	.000
Heating method * salt	.094	6	.016	4.338	.003
Error	.101	28	.004		
Total	61.451	42			
Corrected Total	5.837	41			

a. R Squared = .983（Adjusted R Squared = .975）

Table S11. Tests of Between-Subjects Effects

Dependent Variable: Hydrogen bonds

Source	Type III Sum of Squares	df	Mean Square	F	Sig
Corrected Model	.304[a]	13	.023	16.525	.000
Intercept	3.780	1	3.780	2668.235	.000
Heating method	.058	1	.058	40.901	.000
salt	.234	6	.039	27.576	.000
Heating method * salt	.012	6	.002	1.411	.246
Error	.040	28	.001		
Total	4.124	42			
Corrected Total	.344	41			

a. R Squared = .885（Adjusted R Squared = .831）

Table S12. Tests of Between-Subjects Effects

Dependent Variable: Hydrophobic interactions

Source	Type III Sum of Squares	df	Mean Square	F	Sig
Corrected Model	32.822[a]	13	2.525	445.169	.000
Intercept	100.750	1	100.750	17764.494	.000
Heating method	.104	1	.104	18.338	.000
salt	32.560	6	5.427	956.851	.000
Heating method * salt	.157	6	.026	4.625	.002
Error	.159	28	.006		
Total	133.730	42			
Corrected Total	32.980	41			

a. R Squared = .995（Adjusted R Squared = .993）

Table S13. Tests of Between-Subjects Effects

Dependent Variable: Disulfide bonds

Source	Type III Sum of Squares	df	Mean Square	F	Sig
Corrected Model	160.472[a]	13	12.344	8.144	.000
Intercept	12294.232	1	12294.232	8110.855	.000

Heating method	44.723	1	44.723	29.505	.000
salt	107.857	6	17.976	11.859	.000
Heating method * salt	7.891	6	1.315	.868	.531
Error	42.442	28	1.516		
Total	12497.146	42			
Corrected Total	202.913	41			

a. R Squared = .791（Adjusted R Squared= .694）
